# Improvements in Neonatal and Childhood Medical Care – Perspective from the Balkans

**DOI:** 10.3389/fpubh.2015.00206

**Published:** 2015-08-27

**Authors:** Vesna Velickovic, Aleksandra Simovic, Gordana Lazarevic, Marija Lazarevic, Mihajlo Jakovljevic

**Affiliations:** ^1^Clinic for Pediatrics, University Clinical Centre Kragujevac, Kragujevac, Serbia; ^2^Department of Pediatrics, Faculty of Medical Sciences, University of Kragujevac, Kragujevac, Serbia; ^3^Faculty of Medical Sciences, University of Kragujevac, Kragujevac, Serbia; ^4^Health Economics & Pharmacoeconomics, Faculty of Medical Sciences, University of Kragujevac, Kragujevac, Serbia

**Keywords:** neonatal care, pediatrics, SEE, Serbia, health reform, childhood, Balkans, outcomes

## Traditional Childhood Medical Care Provision – Legacy of Former Yugoslavia

Health services covering needs of pregnant women, newborns, infants, and preschool and school-age children in Serbia, are provided according to the plan of the compulsory health insurance (1). Such insurance premiums include the implementation of organized screening in health institutions of secondary and tertiary levels (prenatal examinations, mandatory screening for phenylketonuria and hypothyroidism, audiometric screening, ophthalmologic screening for retinopathy of prematurity, ultrasound screening for developmental dysplasia of the hip, central nervous system, etc.).

Within the primary health care of newborn infants and infants, regular visits of the attending nurse are mandatory after discharge from the maternity ward. In later period, the chosen doctor does systematic and regular check-ups [complete blood count (CBC) in the age of 6 months, 2 years, and 4 years] and follows the growth and psychomotor development of the child. In preschool and school, medical examinations are performed in dispensary (every other year). Chosen physician is obliged to refer to the additional examinations (speech therapist, dentist, physiatrists, ophthalmologist, and otolaryngologist) in the age of 4 years and before school enrollment. These screenings are used for early detection of functional disorders, in order to carry out structural analysis and preventive interventions to preserve the health of the youngest population in Serbia.

At the same time, the protection from infectious diseases is carried out by vaccination, according to mandatory immunization calendar by age groups. This procedure is consistent with the recommendations of the Republic Institute for Health Insurance and European regional strategy “Health for All.” National public health strategy to cope with communicable diseases is funded by the Republic Institute for Health Insurance (2).

Pregnant women and children in Serbia have the right to regular dental care from the compulsory health insurance (“health booklet”), within the framework of primary health care – the principle of the chosen dentist (3). In the preschools and schools, preventive dental examinations are periodically carried out, with immediate health and educational work with children. The aim is to adopt healthy eating habits, improving oral hygiene, and early caries prophylaxis (i.e., application of fluoride, sealants, etc.).

## Transitional Success Story – Improved Outcomes in Neonatal Care Since the End of 1990s

In the last 15 years, in Serbia, the number of children born in a marriage, properly declines, while their number is in inverse relation to the age of mothers, more exactly, the number of women who give birth after 35 years has been rising (4) (see Table 1). Mostly, that is the case of highly educated women, whose postponing of motherhood is justified by progress in their careers and late finding of adequate partner. Also, the number of single mothers grows, due to the increased number of divorces and poor socio-economic situation in Serbia (5).

**Table 1:** **Selected demographic, child health indicators and fertility-related health care resources (midwife capacity) in Serbia in 1998 and 2012 (HFA-DB)**.*

	1998 or closest year available	2012 or closest year available
Contraceptive use among currently married women aged 15–49 (%), any method	58.7^2000^	60.8^2010^
Midwives (PP) per 100 000	35.53^2003^	36.17
Number of midwives (PP)	2658^2003^	2604
Proportion (%) of births attended by skilled health personnel	98.1^2002^	99.7^2010^
% of all live births to mothers aged under 20 years	12.8	5.59
% of all live births to mothers aged 35+ years	12.72	14.07
% of live births weighing 2500 g or more	95	94
Cesarean sections per 1000 live births	79.81^2000^	267.78
Congenital anomalies per 100 000 live births	2449.4^2006^	5584.55
Births with Down’s syndrome per 100 000 live births	54.93^2006^	31.22
Fetal deaths per 1000 births	5.58	5.47
Perinatal deaths per 1000 births	12.44	6.62
Abortions per 1000 live births	573.75^2000^	302.35
Abortions per 1000 live births, age under 20 years	189.66^2000^	222.75
Abortions per 1000 live births, age 35+ years	2429.72^2000^	746.99

*Data Source: European health for all database (HFA-DB) released by World Health Organization Regional Office for Europe.

On the contrary, the number of abortions in the subpopulation of women over 35 years is in significant decline [almost three times lower compared to 1998 (Table 1)], probably as a result of improved popular education concerning contraception, in relation to underage pregnant women. Although according to the National Institute for Public Health “Dr. Milan Jovanovic Batut,” the number of adolescent pregnancies declined starting from 2009, the number of teenage abortions according to data from 2012 is still alarming (about 223 per year) (6).

These data suggest that the level of sexual culture among teenagers in Serbia remains low, due to lack of awareness about safe methods of contraception and fear that contraception will cause health endanger (real risk of thromboembolic complications at pregnant women under 20 years is decently low), high cost of some contraceptive methods, shame, etc. Encouraging sign is the reduction of perinatal mortality in recent years (Table 1). This data cannot be explained by increasing qualified health personnel capacity (for about 1.6:100,000, according to data from Table 1), but rather with increasing frequency of cesarean section.

Empirically, we know that after prematurity, the leading reasons for perinatal morbidity and mortality are asphyxia and systemic infections. Having in mind that obstetric interventions (forceps and vacuum extraction) are often followed by such complications, we believe that more frequent cesarean section surgery would contribute to significantly reduced overall perinatal mortality (7).

Better prevention of prematurity and treatment of respiratory diseases is mostly attributable to the prenatal application of glucocorticoids, postnatal surfactant application, and less invasive high frequency ventilation devices. The mortality caused by serious grade respiratory distress syndrome (RDS), as the leading risk in premature infants, has been reduced.

Besides, fetal mortality in Serbia still exists (Table 1) and for worrying is the fact that the number of newborn children with congenital anomalies in 2012 was doubled compared to 1998. This can be explained by increased number of older pregnant women (8). On the other hand, after latency period of two decades since the civil wars of Yugoslavia and depleted uranium bombings, population is faced with susceptible consequences of environmental pollution (9, 10).

Taking into consideration the aforementioned facts, we can conclude that pregnant women and children remain particularly vulnerable group. This is reflected in currently endangered population health status in Serbia, primarily indicated by growing impact of malignant diseases among younger age groups (11).

It is interesting, that despite the current trend of giving birth in late ages, there is a certain decrease in the birth of children with Down syndrome. These results suggest that early use of screening tests (“double,” “triple” test, amniocentesis, cordocentesis, fetal ultrasound expert, etc.), especially in women after 35 years, can contribute to the reduction of such perinatal morbidity and perinatal mortality rate. Therefore, we believe that more substantial financial resources should be directed toward improving cost-effective prenatal Down syndrome screening (12).

## Vulnerabilities of Contemporary Early Childhood Medical Care in Serbia

Despite obvious progress in the provision of medical care services within the national health system of Serbia, there are still some ongoing challenges (13). Among high-income European countries, primary health care establishments resolve at least 75% of health problems, such as 84% reported in the United Kingdom (14). In the former Yugoslavia, the same proportion rarely exceeded 50%, which resulted in redirection of significant number of adults and pediatric patients alike to the polyclinic and hospital health care (15).

Very often, waiting time for specialist examinations or necessary radiology diagnostics (16) and therapeutic procedures could range from several weeks up to few months (17). So, the patients are often forced to carry them out-of-pocket in private health institutions (18). Thus, health care costs are increasing and occasionally does not seem to provide adequate gains visible in key population health indicators. This public perception is present in most of the countries originating from the former Yugoslavia (19).

As the published evidence suggests, on the cost of insurance, residents in Serbia may have lower affordability of novel medicines compared to the patients in the surrounding countries (20). The positive list of publicly reimbursed medicines, there are significantly less innovative medicaments (21, 22). Budget share of pharmaceuticals in Serbia was ∼742 million € in 2012 out of 1847 million € total public health expenditure available (23). At the same time, few other countries of the South East Europe region (SEE) succeeded to allocate significantly higher amount (24).

In juvenile gynecology, currently topical issue is the unavailability of many modern methods of contraception (such as progestin pills, depot injections, implants, etc.), or their relative high price. Also, sex education is not adequately adapted to the age of teenagers, medically based, or psychologically supported. Therefore, the number of teenage abortions in Serbia, compared to the countries in the region, continues to be comparably high.

According to the European Health for All Database latest 2013 official release (25), neonatal mortality in Western European countries is only 2:1000 live births (Austria and Belgium). In Serbia, perinatal mortality is still high and approximately corresponds to data from Albania (7:1000) and Bulgaria (6:1000). At the same time, the health care staff capacity in Serbia is similar to in the one of Bosnia and Bulgaria (≈100:100,000) (26).

As for the immunoprophylaxis program, according to the Institute for Public Health “Dr. Milan Jovanovic Batut,” the number of vaccinated children in the first and second year of life in 2012 was below target of 95%, except for BCG and DTP3. Such result is probably the lowest one in the last 20 years. Interruptions in the continuity of immunization implementation in 2012, due to vaccines market shortages and weaker response of parents due to insufficient information, jeopardized the sustainability of the achieved outcomes.

## Core Opportunities for Further Build-Up of Neonatal Care Capacities

In order to prevent unwanted teenage pregnancies in Serbia, the introduction of sex education in teaching units might have very important role, having in mind that according to polls, less than 5% of teenage girls use contraception (27). By reducing the number of unwanted pregnancies at teenage girls, their reproductive health would be preserved and the number of live born children would be increased.

On the other hand, if the trend of giving birth in later living period continues, we can expect growth *in vitro* fertilization, high-risk pregnancies, premature births, and fetal mortality, as well. In order to achieve further decrease of perinatal morbidity and mortality, it is necessary to improve access to the novel medical technologies. It must be simultaneously accompanied by stronger investment into health personnel education, acquisition of medical equipment and more effective management of hospital facilities. Continuing professional training of health neonatal intensive care professionals bears particular significance. Capital investment in the equipment for neonatology centers that could be transferred “toward the patient” to provide emergency care is essential. It assumes procurement of portable incubators, monitors, infusion pumps, portable respirators, and associated intensive care appliances (28).

Another, perhaps more realistic option would be the training of staff in local maternity hospitals to carry out an adequate short-term patient transfer to the specialized referral facilities, and thereby reduce unnecessary engagement of highly educated personnel, out of neonatal units. Unfortunately, the current situation shows that most countries in the SEE region allocate more money for health care then Serbia. Density of clinical physicians in Serbia is still below the European average. On the other hand, there are approximately 2000 physicians, 1200 dentists, 400 pharmacists, and almost 15,000 secondary vocational staff, currently unemployed.

Probably the most cost-effective and feasible solutions to improve neonatal care quality and outcomes in Serbia would be faster pace of replacement of senior staff approaching retirement with younger residency training and specialist physicians, expanded health insurance coverage, wiser resource allocation as well as strengthening of private-owned medical care facilities network. Whether complex transitional health care reform going on over two decades in the region shall make a success story or a lost historical opportunity remains to be seen.

## Conflict of Interest Statement

The authors declare that the research was conducted in the absence of any commercial or financial relationships that could be construed as a potential conflict of interest.
